# Migration experiences and reported commercial and non-commercial sexual behaviors among newly diagnosed HIV infections in China: a cross-sectional study

**DOI:** 10.1186/s12879-023-08333-6

**Published:** 2023-06-01

**Authors:** Yuyin Zhou, Yan Luo, Feng Cheng, Huatang Zeng, Liqun Wu, Liangmin Gao, Junfang Xu

**Affiliations:** 1 Center for Health Policy Studies, School of Public Health, Zhejiang University School of Medicine, 866 Yuhangtang Rd, 310058 Hangzhou, China; 2 Department of Pharmacy, Second Affiliated Hospital, Zhejiang University School of Medicine, Hangzhou, China; 3 Shenzhen Pingshan District Center for Disease Control and Prevention, Shenzhen, China; 4 Hangzhou Center for Disease Control and Prevention, Hangzhou, China; 5 Vanke School of Public Health, Tsinghua University, Beijing, China; 6 Institute for Healthy China, Tsinghua University, Beijing, China; 7 Shenzhen Health Development Research and Data Management Center, Shenzhen, China; 8 Institute for International and Area Studies, Tsinghua University, Beijing, China

**Keywords:** Migration, HIV, Sexual behavior, Health policy

## Abstract

**Background:**

Migration is known to influence human health. China has a high migration rate and a significant number of people who are HIV-positive, but little is known about how these factors intersect in sexual risk behaviors.

**Objective:**

This study aimed to explore sexual risk behaviors between migrants and non-migrants among newly diagnosed HIV infections, and assess the changes of sexual risk behaviors with length of stay in the current city of migrants.

**Methods:**

A cross-sectional questionnaire was conducted among people newly diagnosed with HIV from July 2018 to December 2020 who lived in Zhejiang Province. In the study, sexual risk behaviors included having multiple sexual partners and unprotected sexual behaviors (in commercial sexual behaviors, non-commercial sexual behaviors, heterosexual behaviors, and homosexual behaviors). Binary logistic regression models were employed to explore the influencing factors of sexual risk behaviors, measured by multiple sexual partners and unprotected sexual partners.

**Results:**

A total of 836 people newly diagnosed with HIV/AIDS were incorporated in the study and 65.31% (546) were migrants. The percentages of non-commercial sexual behaviors among migrants were statistically higher than those of non-migrants. Commercial heterosexual behavior was higher among non-migrants compared with migrants. The proportion of study participants having unprotected sexual behaviors and multiple sexual partners with commercial/non-commercial partners was both higher among migrants compared with non-migrants. Among migrants, the likelihood of sexual risk behaviors in both commercial and non-commercial sex increased in the first 3 years and reduced after 10 years. Compared with non-migrants, migrants were statistically associated with multiple sexual partners [*P* = .007, odds ratio (OR) = 1.942]. However, migrants did not exhibit a significant difference in unprotected sexual behaviors compared with non-migrants. In addition, migrants aged between 18 and 45 years who relocated to the current city in the past 2–3 years tended to have multiple sexual partners (*P* < .05).

**Conclusions:**

People newly diagnosed with HIV engaged in different sexual risk behaviors among migrants and non-migrants and more attention should be paid to migrants. For non-migrants, it is urgent to promote the prevention of commercial sexual behaviors. For migrants, prevention of non-commercial sexual behaviors and universal access to health care especially for new arrivals who migrated to the current city for 2–3 years are needed. Moreover, sexual health education and early HIV diagnosis are necessary for the entire population.

## Background

As a major global public health issue, approximately 38.4 million people worldwide were living with HIV (human immunodeficiency virus) at the end of 2021, and a significant number of new infections are reported each year (1.5 million globally in 2021) [1]. Migration has been proved as a potential risk factor for this significant rise in new HIV infections because of biological, socio-economic, and structural factors [2–5]. For example, evidence from the USA, Russia, and the Netherlands all reported that migrants were vulnerable to HIV infection with sexual risk behaviors and tended to become the “bridge population” between destination country and home country [6–11]. For the measurement of sexual risk behavior, some studies used recent sexually transmitted infection or HIV infection as the outcome measure among men who have sex with men, while other studies used unprotected anal sex as the measurement, which included position (i.e., insertive or receptive), HIV status (i.e., positive, negative, or unknown), and partner type (e.g., primary or casual) [12, 13]. For heterosexual adults, sexual risk behavior has been defined as both inconsistent condom use and having multiple sex partners in the past 12 months [14, 15]. Usually, homosexual behaviors with condoms are regarded as low sexual risk behaviors, and high sexual risk behaviors include heterosexual/homosexual behaviors without condoms [16]. In addition, having multiple sexual partners increases the sexual risk [17, 18]. Specifically, sexual risk behavior was defined as sexual intercourse with a casual or new main partner without a condom or without a HIV test for those partners who had ever had intercourse [19].

In China, the economic and social development provide conditions for internal migration, with a significant number of people moving from rural to urban areas, under-developed to developed areas, and western to eastern areas in search of wealth or to seek job opportunities [20]. The 2020 population census of China revealed that there were 375.82 million migrants, which accounted for 26.62% of the total population [21, 22]. Report on China’s Migrant Population Development 2018 showed that 26.0% of the migrant population had at least one infectious disease, and HIV/AIDS accounted for a high proportion [23]. A meta-analysis in China found the incidence rate of HIV among the migrant population is 1.86‰ from 2006 to 2016 [24]. As a southeast coast province, Zhejiang is one of the most developed areas in China, attracting a substantial number of migrants every year (e.g., 6,938,805 individuals moved to Zhejiang during 2010–2020). In addition, the absence of the supervision of family and friends, a sense of anonymity, and an inadequate HIV risk perception offer much sexual freedom, which may result in HIV infection [25–27]. Moreover, migration is instrumental in the spread of HIV, which may transmit HIV from sexual partners in their current city to their partners living at home [28, 29]. Therefore, the large-scale migration in China also has the potential to rapidly transform scattered local HIV outbreaks to an epidemic that is more national in scope.

Whether undertaking “external migration” (i.e., migrating from one country to another) or “internal migration” (i.e., migrating from one region to another in the same country), migrants are regarded as a risk population for infection with HIV/AIDS [11, 30–32]. However, most studies to date have focused on the general difference of sexual risk behaviors and HIV-related knowledge between HIV-negative migrants and non-migrants [33, 34]. Moreover, evidence is limited regarding whether the risk differs among various sexual behaviors (e.g., between commercial and non-commercial sexual behaviors) and people with different migration experiences (e.g., length of stay in the current city); such information is important for the design of targeted public health interventions. Consequently, this study aimed to explore whether sexual behaviors—especially commercial and non-commercial sexual behaviors—differ between migrants and non-migrants with new HIV infections, and whether sexual risk behaviors differ with the length of stay in the current city for migrants with newly diagnosed HIV infections.

## Methods

### Participants and data collection

A cross-sectional survey was conducted among people newly diagnosed as HIV-positive who lived in Zhejiang Province (e.g., Hangzhou, Ningbo, Taizhou, Wenzhou, Jiaxing, Huzhou, Shaoxing, and Jinhua). More than 1000 people are diagnosed with HIV each year in Zhejiang Province [35]. Although the HIV/AIDS (acquired immunodeficiency syndrome) epidemic in Zhejiang Province is not highly prevalent compared with other provinces in China (e.g., Yunnan and Guangxi), migrants accounted for 70% of all newly diagnosed HIV infections in Zhejiang Province [36]. Therefore, we selected Zhejiang Province to explore migration experiences and reported commercial and non-commercial sexual behaviors among newly diagnosed HIV infections.

All people with HIV diagnosed between July 2018 and December 2020 in Hangzhou were invited to participate in the questionnaire survey. The questionnaire was a paper-based structured questionnaire in Chinese. The questionnaire was designed based on the guidelines of intervention work for the prevention of HIV/AIDS issued by the Chinese Center for Disease Control and Prevention and our previous study [37, 38], but some questions were amended to meet the purpose of this study. The survey was conducted when they were diagnosed with HIV positive in the HIV-designated hospitals, and the participants were informed that they could withdraw at any time following the voluntary principle. Staff with experience in AIDS prevention and control from China CDC conducted the survey, and each participant took approximately 15 min to complete the questionnaire. The survey collected information on basic characteristics (e.g., gender, age, monthly salary, marital status), migration experience (e.g., registered residence, current living city, length of stay in the current city), and sexual risk behaviors. The sample size was calculated based on 1.86‰ HIV diagnosed risk among immigrants according to a previous study [24], which requires at least 701 participants. We also estimated the sample size based on 5–10 times the number of questions in the questionnaire, which requires 740 respondents. To maximumly select a representative sample from the population, a total of 836 newly diagnosed people living with HIV/AIDS were incorporated into the study.

### Definition and measurements

In the study, migration was defined as the adults who leave the home city where their registered residence is located and live in another city for the purpose of work or other reasons [39]. Participants were grouped by regions (i.e., eastern, central and western regions) and economic level of their registered residence, as evaluated by per capital gross domestic product (GDP) (i.e., developed and less-developed regions) [40]. Sexual behaviors included commercial sexual behaviors and non-commercial sexual behaviors. Commercial sexual behaviors represented paying for sexual behaviors, which included commercial heterosexual behaviors and commercial homosexual behaviors. Non-commercial sexual behaviors represented sexual behaviors with partners without payment, and included non-commercial heterosexual temporary sex (i.e., having sexual behaviors with strangers or acquaintances for once), non-commercial heterosexual fixed sex (i.e., having sexual behaviors with spouses or ongoing partners), and non-commercial homosexual behaviors [41]. Sexual risk behavior is defined as having multiple sexual partners (i.e., engaging in sexual behaviors with more than one partner) or unprotected sexual behaviors (i.e., engaging in sexual behaviors without condoms). In our study, commercial and non-commercial sexual behaviors were asked conducting in all past years before diagnosis, and having multiple sexual partners or unprotected sexual behaviors were asked conducting in one year before diagnosis. Either having multiple sexual behaviors in commercial or non-commercial sexual behavior was defined as having multiple sexual behaviors. For the frequency of condom use, the answers were classified as “never”, “sometimes”, and “everytime”. Participants who choose “everytime” were defined as having no unprotected sexual behavior, and choose “never” or “sometimes” were defined as having unprotected sexual behavior.

### Statistical analysis

A description method, including frequency, percentage, and mean ± SD, was used to describe the basic demographic characteristics and sexual risk behaviors of people newly diagnosed with HIV. Condom using, multiple sexual partners, and the probability of engaging in sexual risk behaviors were analyzed for migrants and non-migrants, and by length of stay in the current city for migrants. Binary logistic regression models were employed to explore the influencing factors of sexual risk behaviors, measured by multiple sexual partners and unprotected sexual partners. The following independent variables were incorporated into the analysis: migrant experience, age, gender, monthly salary, length of stay in the current city, registered residence by region, and registered residence by economic level. The statistical software SPSS 23.0 (IBM, Armonk, NY, USA) was used to analyze all data. Variables with *P* < .05 were considered statistically significant.

### Ethical considerations

The study protocol and consent procedure were approved and conducted using all relevant guidelines and regulations by the Medical Ethics Committee of Hangzhou Center for Disease Control and Prevention (20,190,712). The informed consent was obtained from all subjects and/or their legal guardian(s). Individual confidentiality was protected as part of the management of individual information and the processing of personal data.

## Results

Table 1 shows the basic characteristics of the study participants who were all newly diagnosed with HIV. In total, 69.86% (584) of the participants were male, the median age was 46 (32, 57) years, the median monthly salary was 3500 [1600, 5000] RMB, and 73.92% (618) of the participants were married. Among the participants, 35.77% (299) were from eastern regions, 10.05% (84) were from central regions, and 11.60% (97) were from western regions. For economic level, 32.30% (270), 13.16% (110), and 11.96% (100) of participants were from developed regions, medium-developed regions, and less-developed regions, respectively. As for the self-perceived transmission way, 22.97% (192) thought it was commercial heterosexual behavior, followed by non-commercial heterosexual temporary sex (18.18%, 152), heterosexual fixed sex (12.56%, 105), non-commercial homosexual behaviors (1.32%, 11), commercial homosexual behaviors (0.12%, 1), and blood infection (0.12%, 1). Among all participants, 65.31% (546) were migrants and 34.69% (290) were non-migrants. For migrants, 38.35% (163) had been living in their current city for 0–1 year, 36.47% (155) for 2–9 years, and 25.18% (107) for ≥ 10 years.

**Table 1:** The basic characteristics of newly diagnosed HIV people

Items	Number	Percentage (%)
Gender		
Male	584	69.86
Female	246	29.43
Missed	6	0.72
Age (Median, Interquartile Range)		46 (32, 57)
Monthly salary (Median, Interquartile Range)		3500 (1600, 5000)
Marital status		
Married	618	73.92
Non-married	195	23.33
Missed	23	2.75
Self-perceived transmission way		
Commercial heterosexual behavior	192	22.97
Non-commercial heterosexual temporary sex	152	18.18
Non-commercial heterosexual fixed sex	105	12.56
Commercial homosexual behaviors	1	0.12
Non-commercial homosexual behaviors	11	1.32
Drug using	0	0
Blood infection	1	0.12
Don not know	11	1.32
Missed	363	43.42
HIV test before diagnosed		
Yes	53	6.33
No	425	50.84
Missed	358	42.82
Migration experience		
Yes	546	65.31
No	290	34.69
Length of stay in the current city among migrants (year)		
0–1	163	38.35
2–3	75	17.65
4–6	53	12.47
7–9	27	6.35
≥10	107	25.18
Missed	121	28.47
Registered residence by region		
Eastern regions	262	31.34
Central and western regions	218	26.08
Missed	356	42.58
Registered residence by economic level		
Developed regions	283	33.85
Less-developed regions	197	23.56
Missed	356	42.58

The sexual risk behaviors between migrants and non-migrants are shown in Table 2. The percentages of non-commercial sexual behaviors among migrants were statistically higher than those of non-migrants (39.93% vs. 28.28% for non-commercial heterosexual temporary sex, and 26.56% vs. 15.52% for non-commercial heterosexual fixed sex). Commercial heterosexual behavior was higher among non-migrants (51.03%, 148) compared with migrants (39.93%, 218) (*P* = .002).

**Table 2:** Migration and sexual risk behaviors before diagnosed with HIV

Items	Migrant		Non-migrant		*P* value
	Number	Percentage (%)	Number	Percentage (%)	
Drug using					0.616
Yes	17	3.11	9	3.10	
No	529	96.89	281	96.90	
Commercial heterosexual behavior					*0.002*
Yes	218	39.93	148	51.03	
No	328	60.07	142	48.97	
Non-commercial heterosexual temporary sex					*0.001*
Yes	218	39.93	82	28.28	
No	328	60.07	208	71.72	
Non-commercial heterosexual fixed sex					*< 0.001*
Yes	145	26.56	45	15.52	
No	401	73.44	245	84.48	
Homosexual behaviors					0.077
Yes	19	3.48	4	1.38	
No	527	96.52	286	98.62	
Commercial homosexual behaviors					0.466
Yes	1	0.18	0	0	
No	545	99.82	290	100	
Non-commercial homosexual behaviors					0.909
Yes	10	1.83	5	1.72	
No	537	98.17	286	98.28	

**Fig. 1:**
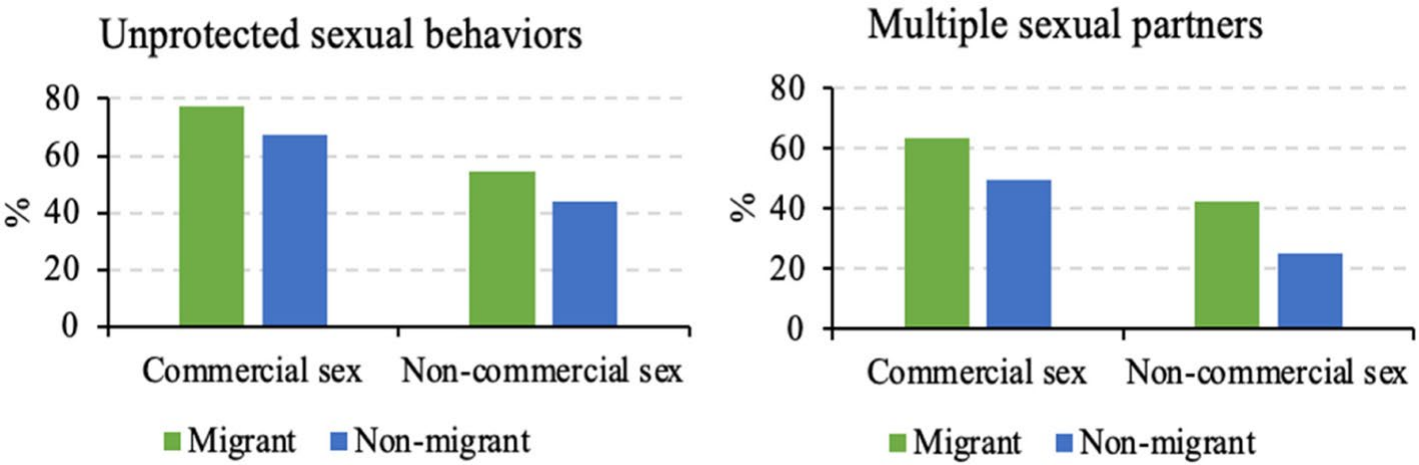
Unprotected sexual behaviors and multiple sexual partners between migrant and non-migrant

The proportion of study participants having unprotected sexual behaviors and multiple sexual partners with commercial/non-commercial partners was both higher among migrants compared with non-migrants (Fig. 1).

**Fig. 2:**
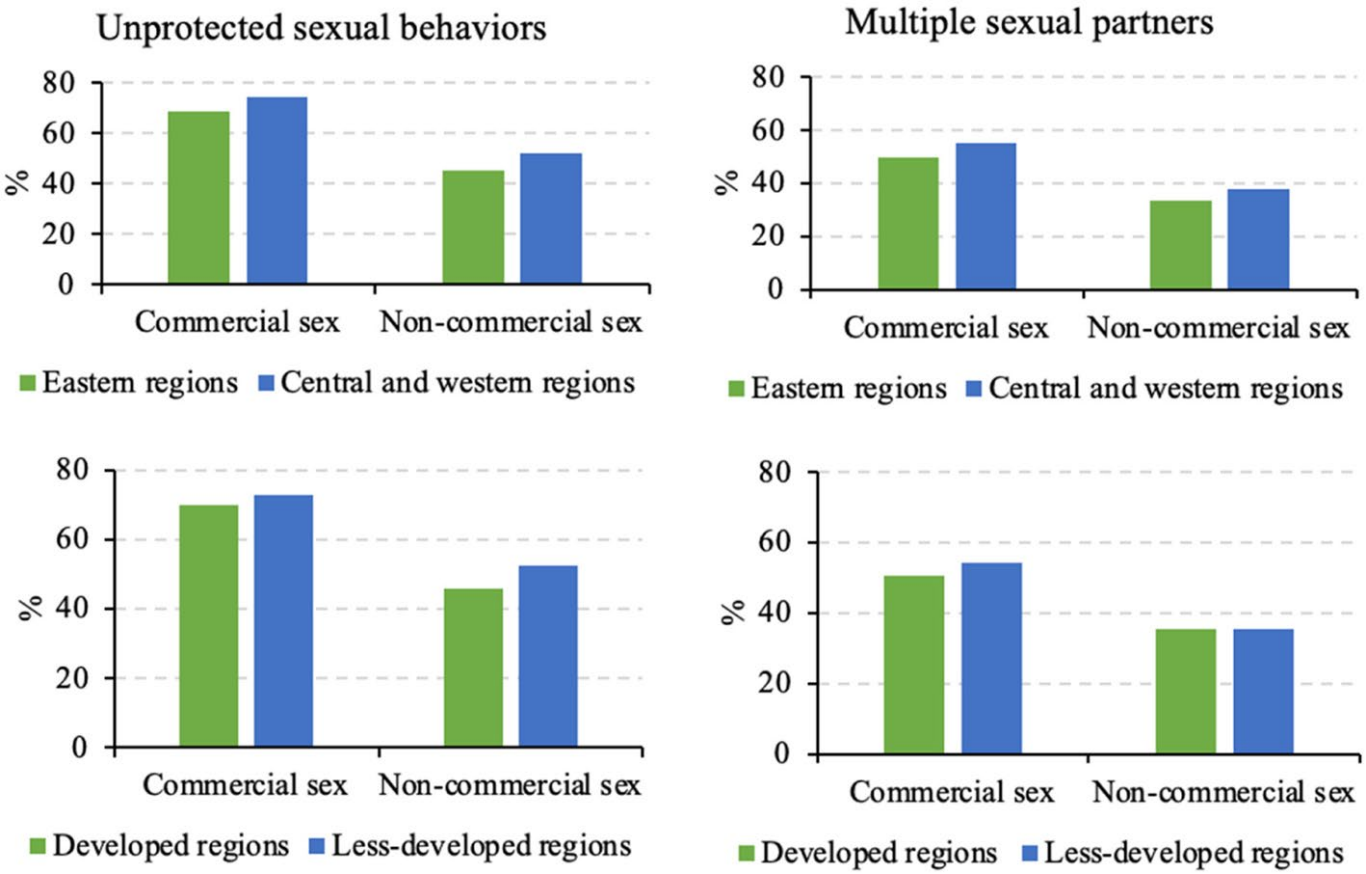
Unprotected sexual behaviors and multiple sexual partners by regions and economic levels

Both in commercial and non-commercial sex, the percentage who having unprotected sexual behaviors was higher among people from central and western regions or less-developed regions compared with those from eastern or developed regions. In terms of having multiple sexual partners, the percentage of people from central and western regions was higher in commercial and non-commercial sex compared with that of people from eastern regions (Fig. 2). However, the results of logistic models showed that there was no significant difference between regions and economic levels on unprotected sexual behaviors and multiple sexual partners (*P* > .05).

**Fig. 3:**
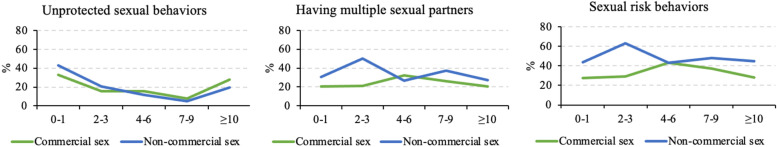
Unprotected sexual behaviors, having multiple sexual partners, and sexual risk behaviors among migrants by length of time living in the current city

Moreover, among migrants, the likelihood of having unprotected sexual behaviors reduced and then increased with the length of time living in the current city. Generally, the percentage of having multiple sexual partners tended to increase initially and then decrease with the length of time living in the current city. For commercial sex, the likelihood of sexual risk behaviors increased in the first 3 years (from 27.44%, 45 to 28.95%, 26) of living in the current city, then reduced to 28.16% (29) after 10 years. For non-commercial sex, the likelihood increased in the first 3 years (from 43.90%, 72 to 63.16%, 48), then reduced to 44.67% (46) after 10 years (Fig. 3).

The results of logistic models showed that migrants were statistically associated with multiple sexual partners (*P* = .007, odds ratio (OR) = 1.942) compared with non-migrants (Table 3). However, migrants did not exhibit a significant difference in unprotected sexual behaviors compared with non-migrants, and registered residence by regions and economic levels also had no significant effect on sexual risk behaviors. Males were likely to engage in unprotected sexual behaviors compared with females. In addition, migrants aged between 18 and 45 years and living in the current city for 2–3 years tended to have multiple sexual partners (*P* < .05).

**Table 3:** The influencing factors of sexual risk behaviors among total participants and migrants

Items	Multiple sexual partners (No = 0, Yes = 1)				Unprotected sexual behaviors (No = 0, Yes = 1)			
	Total		Migrant		Total		Migrant	
	*P* value	Adjusted OR (95%CI)	*P* value	Adjusted OR (95%CI)	*P* value	Adjusted OR (95%CI)	*P* value	Adjusted OR (95%CI)
Migration experience								
Migrant	***0.007***	1.942(1.204,3.132)	*-*	*-*	0.218	1.338(0.842,2.126)	*-*	*-*
Non-migrant (reference)			*-*	*-*			*-*	*-*
Age (year)								
18–45	***0.003***	2.265(1.312,3.908)	***0.007***	2.943(1.344,6.444)	***0.001***	2.441(1.464,4.071)	***0.013***	2.591(1.221,5.499)
46–59	***0.047***	1.79(1.008,3.178)	0.481	1.359(0.579,3.189)	0.101	1.557(0.917,2.644)	0.454	1.361(0.607,3.051)
≥ 60 (reference)								
Gender								
Male	0.199	1.332(0.860,2.064)	0.995	1.002(0.542,1.852)	***0.008***	1.775(1.159,2.719)	0.356	1.333(0.724,2.456)
Female (reference)								
Monthly salary (RMB)								
0-1000	0.058	0.515(0.259,1.023)	0.189	0.527(0.202,1.371)	0.936	1.029(0.518,2.042)	0.656	0.806(0.312,2.080)
1001–4000	0.212	0.667(0.353,1.260)	0.196	0.569(0.242,1.338)	0.594	1.192(0.625,2.276)	0.848	1.086(0.466,2.533)
4001–8000	0.470	0.786(0.410,1.509)	0.638	0.807(0.331,1.971)	0.400	1.332(0.684,2.594)	0.485	1.378(0.560,3.388)
≥ 8001(reference)								
Length of stay in the current city among migrants (year)								
0–1	-	-	0.283	1.47(0.728,2.968)	-	-	0.701	1.146(0.571,2.303)
2–3	-	-	***0.006***	3.209(1.387,7.424)	-	-	0.208	1.724(0.739,4.023)
4–6	-	-	0.395	1.463(0.608,3.521)	-	-	0.695	0.837(0.345,2.033)
7–9	-	-	0.893	1.087(0.321,3.679)	-	-	0.756	0.829(0.255,2.696)
≥ 10 (reference)								
Registered residence by region								
Eastern regions	0.562	0.784(0.345,1.784)	0.901	0.938(0.346,2.548)	0.682	1.189(0.519,2.726)	0.575	1.332(0.489,3.631)
Central and western regions (reference)								
Registered residence by economic level								
Developed regions	0.280	1.567(0.694,3.540)	0.761	1.156(0.454,2.943)	0.651	0.827(0.363,1.883)	0.413	0.676(0.264,1.726)
Less-developed regions (reference)								

## Discussion

In this study, people newly diagnosed with HIV engaged in different sexual risk behaviors among migrants and non-migrants. Migrants who were HIV-positive accounted for 65.31% (546) of the study participants. Numerous studies have demonstrated positive associations between migration and the spread of HIV [20, 29, 42], and migrants also tended to have a higher incidence rate of delayed HIV diagnosis [43]. For example, a study in South Africa indicated that the economic marginalization and social isolation led to migrants with multiple sexual partners in order to escape from solitude and get rid of the anxiety about work and life [44]. A previous study in Netherlands showed that about 50% of the migrants with HIV were late diagnosed [45]. Moreover, based on the healthy immigrant effect, migrants frequently have better health compared with local residents at the outset. However, with the pressure of cultural differences, and the process of integration into a new environment, this health advantage tends to gradually disappear, which may cause their long-term health to be worse than that of non-migrants [20, 42]. Migrants may have less opportunities to receive health education, including sexual health education offered by local health institutions for permanent residents [46]. Inadequate sexual risk perception may also increase the risk of sexual behaviors [20, 34].

In our study, migrants tended to engage in more non-commercial sexual behaviors, while non-migrants tended to engage in more commercial sexual behaviors. Through unfamiliar and unstable living conditions, migrants face unusual pressures such the absence of company from family and friends, which increases the vulnerability and insecurity of migrants. Such physical and mental insecurity leads to migrants having a need for company and emotional support [15], which may cause them to engage in non-commercial sexual behaviors simply for emotional need. A study in Hangzhou, Zhejiang province found that 65.13% of the newly reported HIV/AIDS cases with non-marital or non-commercial heterosexual transmission from 2017 to 2019 were migrants [47]. Non-migrants usually have better economic conditions, higher sexual socialization, and easier access to the local entertainment venues for social needs, thus non-migrants may be likely to have more commercial sexual behaviors [29]. However, there were also studies showed the high probability among migrants engaging in commercial sex [48], and the increasing rate of commercial sex among unmarried male migrants may be because of the marriage squeeze society (i.e., the relative scarcity of women to men in an area which causes males hardly to be married) of China [31]. Additionally, it can be explained by the accessibility of commercial sex (e.g., low price and acquaintance society) in rural China [49], but this study was conducted in urban China, the poor accessibility may result in fewer commercial sexual behaviors.

Although migrants did not show a significant difference in unprotected sexual behaviors compared with non-migrants, a higher number of migrants had multiple sexual partners compared with the non-migrants. In most cases, migrants live in poor neighborhoods and experience difficulties in applying for a steady and long-term job. A previous study showed that unmarried women living in communities in which temporary and casual work was most prevalent tended to have multiple sexual partners [17]. In Chinese rural areas, the relationship between people is much closer than in urban areas, and the family institution is placed in a significant position [50]. Consequently, when anyone breaks the routine, engaging in such as casual sex or extra-marital sexual relationships, it is likely to become the after-dinner speaking of neighbors in rural areas. However, when people from rural areas migrate to a new place, a sense of sexual freedom may appear [48], which may lead to these kinds of behaviors (i.e., multiple sexual behaviors). Moreover, evidence showed that migration was a male domain, especially among young males who are in a period of active sexual behaviors [29, 34]. These men usually left their established communities and migrated without their wives and family members [29]. It has been reported that unaccompanied married migrant males are likely to have extramarital affairs [51–53]. A prior study in China showed that over 40% of male labor migrants had multiple sexual partners [34]. Correspondingly, individuals migrating with their spouses tend to have a low risk of engaging in casual sex [27]. We also observed that among migrants, new arrivals who moved to the current city for about 2–3 years tended to engage in having multiple sexual partners. A study in Uganda indicated that HIV incidence was highest in the first 2 years after migrating into the destination area regardless of gender, which suggested that migrants did not benefit from HIV-related services in the first 2 years after migration [54]. Nevertheless, some studies showed the opposite conclusion. For instance, European researchers found the longer the length of stay in the destination country, the higher was the probability of infected with HIV after migration [55, 56]. It may result from the differences in the migration experience of the study participants (internal migration vs. external migration) and the economic level of the destination regions (developing country vs. developed country). As discussed above, the pressures caused by instabilities, uncertainties, and isolation among migrants of their relocation may expose them to various sexual risk behaviors [48].

Inequality in China is still a huge barrier to HIV prevention and control, and migrants are tended to be disproportionately affected by HIV [57, 58]. The crucial issue for migrants’ health is not their social characteristics such as poor living conditions or the lack of health awareness, but the institutional problems regarding health security and service provision [59]. The government is supposed to provide universal access to health care to migrants regardless of their length of stay in the current regions and include more anti-HIV drugs into the medical insurance.

Health education is an effective way to enhance knowledge about HIV, regardless of whether people are migrants or non-migrants. UNAIDS proposed that strengthening and expanding community-led HIV prevention services is one of the ten point action plans [60]. Therefore, more HIV education resources need to be allocated to communities with large numbers of migrants, and these resources need to highlight the risk of having multiple sexual partners even though they are not strangers or sex workers. More emphasis should be placed on condom use in sexual behaviors during HIV-related health education. Migrants are more likely to use free condoms which suggest expanding the distribution channels of free condoms may improve the acceptance of condom use [61]. In addition, providing multiple options in health education, including both new and traditional media for migrants is essential [62]. For example, HIV-related knowledge could be advertised on television, in the press, and mass transportation, and it may promote positive community-wide attitudes about HIV [63]. However, though many HIV education activities have been conducted over the years, there are still marked challenges in the early diagnosis and early detection of HIV in China. The perceived stigmatization of HIV [27] means people with sexual risk behaviors may be not willing to be tested in an institutionalized setting, which may delay HIV diagnosis. An alternative approach that is convenient and effectively protects privacy is HIV self-testing, and this should be promoted in communities, where it has the potential to reach every person who has sexual risk behaviors and tends to have the test [64]. Moreover, it is an effective way to incorporate HIV testing into the emergency department in order to enhance the early HIV diagnosis [65, 66].

## Limitations

First, considering the large geographical area, the cultural differences, and the differences in the predominant routes of HIV transmission in different areas of China, our results from only one province cannot fully represent the behavior of other people newly diagnosed with HIV. Also, the time period was July 2018 – December 2020 in the study design, so the results may be limited to extending after 2021. Second, this cross-sectional study cannot explore the causality of migration and sexual risk behaviors. Third, there were some missing responses on prior HIV testing status, length of stay in the current city and it is also important to explore the refusal rate and reasons. Fourth, considering data regarding previous HIV tests were missing, we could not distinguish whether the migration happened before or after the HIV diagnosis. Finally, although the questionnaire was designed based on the guidelines of intervention work for the prevention of HIV/AIDS issued by the Chinese Center for Disease Control and Prevention and previous studies, some questions were amended to meet the purpose of this study. Thus, the validity of the questionnaire needs further testing.

## Conclusion

People newly diagnosed with HIV engaged in different sexual risk behaviors among migrants and non-migrants and more attention should be paid to migrants. For non-migrants, it is urgent to promote the prevention of commercial sexual behaviors. For migrants, prevention of non-commercial sexual behaviors and universal access to health care especially for new arrivals who migrated to the current city for 2–3 years are needed. Moreover, sexual health education and early HIV diagnosis are necessary for the entire population.

## Data Availability

All of the principal data are included in the results. Additional materials with further details may be obtained from the corresponding author.

## References

[1] HIV data and statistics. [https://www.who.int/teams/global-hiv-hepatitis-and-stis-programmes/hiv/strategic-information/hiv-data-and-statistics].

[2] Nöstlinger C, Loos J (2018). Migration patterns and HIV prevention in Uganda. Lancet HIV.

[3] Lee JJ, Yu G (2019). HIV Testing, Risk Behaviors, and fear: a comparison of documented and undocumented latino immigrants. AIDS Behav.

[4] Rodríguez-Álvarez E, Lanborena N, Bacigalupe A, Martin U (2013). Social factors associated with the knowledge about HIV of the immigrants from China, Latin America, the Maghreb and Senegal in the Basque Country (Spain). J Immigr Minor Health.

[5] Nkulu-Kalengayi FK, Ouma AA, Hurtig AK (2022). HIV ended up in second place’ - prioritizing social integration in the shadow of social exclusion: an interview study with migrants living with HIV in Sweden. Int J Equity Health.

[6] Persichino J, Ibarra L (2012). HIV and latino migrant workers in the USA. Ethnic and Racial Studies.

[7] Bronfman MN, Leyva R, Negroni MJ, Rueda CM (2002). Mobile populations and HIV/AIDS in Central America and Mexico: research for action. Aids.

[8] Amirkhanian YA, Kuznetsova AV, Kelly JA, Difranceisco WJ, Musatov VB, Avsukevich NA, Chaika NA, McAuliffe TL (2011). Male labor migrants in Russia: HIV risk behavior levels, contextual factors, and prevention needs. J Immigr Minor Health.

[9] Gras MJ, van Benthem BH, Coutinho RA, van den Hoek A (2001). Determinants of high-risk sexual behavior among immigrant groups in Amsterdam: implications for interventions. J Acquir Immune Defic Syndr.

[10] Del Amo J, Bröring G, Hamers FF, Infuso A, Fenton K (2004). Monitoring HIV/AIDS in Europe’s migrant communities and ethnic minorities. Aids.

[11] Yin Z, Brown AE, Rice BD, Marrone G, Sönnerborg A, Suligoi B, Sasse A, Van Beckhoven  D, Noori T, Regine V (2021). Post-migration acquisition of HIV: Estimates from four European countries, 2007 to 2016. Euro Surveill.

[12] Vosburgh HW, Mansergh G, Sullivan PS, Purcell DW (2012). A review of the literature on event-level substance use and sexual risk behavior among men who have sex with men. AIDS Behav.

[13] Scheer JR, Clark KA, Maiolatesi AJ, Pachankis JE (2021). Syndemic profiles and sexual minority men’s HIV-Risk Behavior: a latent class analysis. Arch Sex Behav.

[14] Brooks RA, Lee SJ, Newman PA, Leibowitz AA (2008). Sexual risk behavior has decreased among men who have sex with men in Los Angeles but remains greater than that among heterosexual men and women. AIDS Educ Prev.

[15] Jirattikorn A, Tangmunkongvorakul A, Musumari PM, Ayuttacorn A, Srithanaviboonchai K, Banwell C, Kelly M (2020). Sexual risk behaviours and HIV knowledge and beliefs of Shan migrants from Myanmar living with HIV in Chiang Mai, Thailand. Int J Migration Health Social Care.

[16] Li LL, Jiang Z, Song WL, Ding YY, Xu J, He N (2017). Development of HIV infection risk assessment tool for men who have sex with men based on Delphi method. Chin J Epidemiol.

[17] Uchudi J, Magadi M, Mostazir M (2012). A multilevel analysis of the determinants of high-risk sexual behaviour in sub-saharan Africa. J Biosoc Sci.

[18] Aidoo-Frimpong G, Agbemenu K, Orom H (2021). A review of Cultural Influences on Risk for HIV and culturally-responsive risk mitigation strategies among african immigrants in the US. J Immigr Minor Health.

[19] Brodbeck J, Matter M, Moggi F (2006). Association between cannabis use and sexual risk behavior among young heterosexual adults. AIDS Behav.

[20] Chen G, Lv J (2005). The Considerationon migrant’s Public Health Management in China. Med Philos.

[21] Bulletin of the Seventh National Census. (No. 2) [http://www.stats.gov.cn/tjsj/tjgb/rkpcgb/qgrkpcgb/202106/t20210628_1818821.html].

[22] Bulletin of the Seventh National Census (No. 8) [http://www.stats.gov.cn/tjsj/tjgb/rkpcgb/qgrkpcgb/202106/t20210628_1818826.html].

[23] National Health Commission. Report on China’s Migrant Population Development 2018[M]. Beijing: China Population Publishing House; 2018: 172.

[24] Qiu HH. Cumulative Meta-analysis of HIV infection, AIDS related behavior characteristics and AIDS related knowledge of the mobile population in China. Jiangxi: Master Nanchang University; 2017.

[25] Qiu P, Yang Y, Wu F, Cao X, Zhao. S, Ma X (2010). Progress and enlightenment of home and abroad researches on mental health of mobile population. Chin Mental Health J.

[26] Detels R, Wu Z, Rotheram MJ, Li L, Guan J, Yin Y, Liang G, Lee M, Hu L (2003). Sexually transmitted disease prevalence and characteristics of market vendors in eastern China. Sex Transm Dis.

[27] Hesketh T, Li L, Ye X, Wang H, Jiang M, Tomkins A (2006). HIV and syphilis in migrant workers in eastern China. Sex Transm Infect.

[28] Hu Z, Liu H, Li X, Stanton B, Chen X (2006). HIV-related sexual behaviour among migrants and non-migrants in a rural area of China: role of rural-to-urban migration. Public Health.

[29] Ajaero CK, Onuh JC, Amoo EO, Adewoyin Y. Contextual correlates of risky sexual behavior among migrant and non-migrant men in Nigeria. SAGE Open 2020;10(2).

[30] Fuller TD, Chamratrithirong A (2009). Knowledge of HIV risk factors among immigrants in Thailand. J Immigr Minor Health.

[31] Yang B, Li S, Wu Z (2016). Accumulative risk of HIV/AIDS among male migrants: a study based on the commercial sex analysis and policy implication. Popul Dev.

[32] Chen S, Cao XB (2020). HIV/AIDS prevention and treatment among foreigners living in China: a research update. Chin J AIDS STD.

[33] Yang CHJ, Tao MX, Li YB, Chai Y, Ning Y, Li L, Xiao Q (2014). Knowledge about AIDS and condom use among migrant populations in Beijing city. Chin J Public Health.

[34] Yang B, Wu Z, Schimmele CM, Li S (2015). HIV knowledge among male labor migrants in China. BMC Public Health.

[35] Fu TY, Wu HC, Lu QB, Ding ZY, Wang XY, Yang K, Wu C, Lin JF (2022). Epidemiological characteristics of notifiable infectious diseases in Zhejiang Province, 2021. Prev Med.

[36] AIDS epidemic slows down. In Zhejiang province, but the proportion of the elderly population rises [https://ori.hangzhou.com.cn/ornews/content/2017-12/01/content_6730842.htm?bsh_bid=1887758535].

[37] Xu J, Luo Y, Dong H, Zhao G (2022). The Effects of Internet exposure on sexual risk behavior among sexually experienced male College students in China: cross-sectional study. JMIR Public Health Surveill.

[38] Xu JF, Wang PC, Cheng F (2020). Health related behaviors among HIV-infected people who are successfully linked to care: an institutional-based cross-sectional study. Infect Dis Poverty.

[39] Hu X, Cook S, Salazar MA (2008). Internal migration and health in China. Lancet.

[40] Jung M (2012). Immigrant workers’ knowledge of HIV/AIDS and their sexual risk behaviors: a Respondent-Driven Sampling Survey in South Korea. Sex Disabil.

[41] Mayanja Y, Mukose AD, Nakubulwa S, Omosa-Manyonyi G, Kamali A, Guwatudde D (2016). Acceptance of treatment of sexually transmitted infections for stable sexual partners by female sex workers in Kampala, Uganda. PLoS ONE.

[42] Hesketh T, Ye X, Li L, Wang H (2008). Health status and access to health care of migrant workers in China. Public Health Rep.

[43] Alvarez-del Arco D, Monge S, Azcoaga A, Rio I, Hernando V, Gonzalez C, Alejos B, Caro AM, Perez-Cachafeiro S, Ramirez-Rubio O (2013). HIV testing and counselling for migrant populations living in high-income countries: a systematic review. Eur J Pub Health.

[44] Jochelson K, Mothibeli M, Leger JP (1991). Human immunodeficiency virus and migrant labor in South Africa. Int J Health Serv.

[45] van Bilsen WPH, Bil JP, Prins JM, Brinkman K, Leyten E, van Sighem A, Bedert M, Davidovich U, Burns F, Prins M (2022). Testing and healthcare seeking behavior preceding HIV diagnosis among migrant and non-migrant individuals living in the Netherlands: directions for early-case finding. PLoS ONE.

[46] Hong Y, Stanton B, Li X, Yang H, Lin D, Fang X, Wang J, Mao R (2006). Rural-to-urban migrants and the HIV epidemic in China. AIDS Behav.

[47] Zhao GZX, Chen JF, Xu K, Wu H (2021). Characteristics of HIV/AIDS cases with non-marital or non-commercial heterosexual transmission in Hangzhou. Prev Med.

[48] Yang XS, Xia GM (2008). Temporary migration and STD/HIV risky sexual behavior: a population-based analysis of gender differences in China. Soc Probl.

[49] Xiao Q, Liu H, Wu B (2020). How Bachelorhood and Migration increase the HIV Transmission Risk through Commercial Sex in China?. AIDS Behav.

[50] Jobin C (1993). Religion, family planning, and sexual behavior. Lancet.

[51] Viadro CI, Earp JA (2000). The sexual behavior of married mexican immigrant men in North Carolina. Soc Sci Med.

[52] Ankomah A, Adebayo SB, Anyanti J, Ladipo O, Ekweremadu B (2013). Determinants of condom use by men in extramarital relationships in Nigeria. HIV AIDS (Auckl).

[53] UNAIDS JUNPoHA (1998). Migration and AIDS. Int migration (Geneva Switzerland).

[54] Olawore O, Tobian AAR, Kagaayi J, Bazaale JM, Nantume B, Kigozi G, Nankinga J, Nalugoda F, Nakigozi G, Kigozi G (2018). Migration and risk of HIV acquisition in Rakai, Uganda: a population-based cohort study. Lancet HIV.

[55] Alvarez-Del Arco D, Fakoya I, Thomadakis C, Pantazis N, Touloumi G, Gennotte AF, Zuure F, Barros H, Staehelin C, Göpel S (2017). High levels of postmigration HIV acquisition within nine european countries. Aids.

[56] Desgrées-du-Loû A, Pannetier J, Ravalihasy A, Gosselin A, Supervie V, Panjo H, Bajos N, Lert F, Lydié N, Dray-Spira R. Sub-saharan african migrants living with HIV acquired after migration, France, ANRS PARCOURS study, 2012 to 2013. Euro Surveill 2015, 20(46). 10.2807/1560-7917.ES.2015.20.46.30065 26607135

[57] The Joint United Nations Programme on HIV/AIDS (2022). Danger: UNAIDS Global AIDS Update 2022.

[58] The Joint United Nations Programme on HIV/AIDS (2021). Global AIDS Strategy 2021–2026 — end inequalities. End AID.

[59] Xiang B. Migration and Health in China: Problems, Obstacles and Solutions. Asian Metacentre for Population and Sustainable Development Analysis. 2003. 10.13140/RG.2.2.26600.21766.

[60] The Joint United Nations Programme on HIV/AIDS (2022). HIV prevention 2025 road map — Getting on track to end AIDS as a public health threat by 2030.

[61] Wang Y, Cochran C, Xu P, Shen JJ, Zeng G, Xu Y, Sun M, Li C, Li X, Chang F (2014). Acquired immunodeficiency syndrome/human immunodeficiency virus knowledge, attitudes, and practices, and use of healthcare services among rural migrants: a cross-sectional study in China. BMC Public Health.

[62] Zhu Z, Guo M, Petrovsky DV, Dong T, Hu Y, Wu B (2019). Age and regional disparity in HIV education among migrants in China: migrants population dynamic monitoring survey, 2014–2015. Int J Equity Health.

[63] Chin JJ, Mantell J, Weiss L, Bhagavan M, Luo X (2005). Chinese and south asian religious institutions and HIV prevention in New York City. AIDS Educ Prev.

[64] World Health Organization. (2018). HIV self-testing strategic framework: a guide for planning, introducing and scaling up.

[65] Tan R, Hugli O, Cavassini M, Darling K (2018). Non-targeted HIV testing in the emergency department: not just how but where. Expert Rev Anti Infect Ther.

[66] Hempling MC, Zielicka-Hardy A, Ellis JP, Majewska W, Fida G (2016). Routine HIV testing in the Emergency Department: feasible and acceptable?. Int J STD AIDS.

